# No evidence of physiological declines with age in an extremely long-lived fish

**DOI:** 10.1038/s41598-021-88626-5

**Published:** 2021-04-27

**Authors:** Derek J. Sauer, Britt J. Heidinger, Jeffrey D. Kittilson, Alec R. Lackmann, Mark E. Clark

**Affiliations:** 1 Institute of Marine Science, University of Auckland, Leigh, 0985 New Zealand; 2 Department of Biological Sciences, North Dakota State University, Fargo, ND 58102 USA; 3 Department of Biology, University of Minnesota Duluth, Duluth, MN 55804 USA

**Keywords:** Physiology, Ageing, Zoology, Ichthyology

## Abstract

Although the pace of senescence varies considerably, the physiological systems that contribute to different patterns of senescence are not well understood, especially in long-lived vertebrates. Long-lived bony fish (i.e., Class Osteichthyes) are a particularly useful model for studies of senescence because they can readily be aged and exhibit some of the longest lifespans among vertebrates. In this study we examined the potential relationship between age and multiple physiological systems including: stress levels, immune function, and telomere length in individuals ranging in age from 2 to 99 years old in bigmouth buffalo (*Ictiobus cyprinellus*), the oldest known freshwater teleost fish. Contrary to expectation, we did not find any evidence for age-related declines in these physiological systems. Instead, older fish appeared to be less stressed and had greater immunity than younger fish, suggesting age-related *improvements* rather than declines in these systems. There was no significant effect of age on telomeres, but individuals that may be more stressed had shorter telomeres. Taken together, these findings suggest that bigmouth buffalo exhibit negligible senescence in multiple physiological systems despite living for nearly a century.

## Introduction

Senescence is characterized by age-related changes that adversely affect physiological function and ultimately reduce fitness^1^. Research in a wide range of species has demonstrated declines in diverse life-history traits in old age consistent with senescence (reviewed by Nussey et al.^2^). Although evidence for senescence is accumulating, there is considerable variation in the rate of senescence among and even within species^2^. Some organisms have extremely slow rates of aging. For example, captive naked mole-rats display minimal changes in physiology or morphology while living more than 28 years, approximately 9 times longer than mice of similar size, and females show no decline in fertility even near the end of their lifespan^3^. In addition, some turtle species display no sign of increased mortality or loss of vigor in old age and older females lay more eggs and have more consistent reproduction than younger females^4^ (although see Warner et al*.*^5^). Species that exhibit extremely slow senescence reveal the notable variation that exists in pace of senescence and highlight the value of studying senescence in slow-aging species.

Studies on senescence often focus on age-related changes in mortality and fecundity without quantifying changes in the function of physiological systems, and the physiological systems that contribute to differences in senescence patterns are not well understood, especially in long-lived vertebrates. Age-related changes to physiological systems appear inconsistent across vertebrates and may depend upon the system that is examined. For example, studies in long-lived seabirds have found little evidence of a decline in immunity^6,7^, stress response^7^, reproduction^8^, or metabolism^9^. However, a clear decline in immune resistance to parasites was found in late-life in Soay sheep (*Ovis aries*)^10^. Few studies have examined how multiple physiological systems change with age. However, because senescence is a complex phenotype, quantifying age-related changes within different physiological systems in wild populations can help us better understand the progression of senescence. Age-related changes in mortality and fecundity inform us about general rates of senescence, but understanding the underlying mechanisms requires studies of age-related changes in physiological systems.

Long-lived fishes are excellent candidates for studying senescence in free-living vertebrates. First, long-lived fishes possess some of the longest lifespans observed within vertebrates^11^, suggesting slow rates of senescence. Second, fish can be accurately aged using hard internal structures (e.g., otoliths), a convenience not offered by many other vertebrates (e.g., mammals, birds, and amphibians). Lastly, teleost fishes comprise about half of all vertebrate species^12^, making them a crucial part of understanding vertebrate evolution and life history. These life history traits make long-lived fish an exceptional model organism for studying senescence in different physiological systems.

Here, we examined the potential effects of age on multiple physiological systems expected to be involved in senescence in bigmouth buffalo (*Ictiobus cyprinellus*). Until recently, bigmouth buffalo life history (e.g., growth rate, reproductive maturation, lifespan) had not been accurately quantified. However, recent research using otoliths (‘earstones’ that can be used to accurately age individual fish) and bomb radiocarbon age validation has demonstrated that bigmouth buffalo can live for at least 112 years, making them the longest-lived freshwater teleost (~ 12,000 species), and the oldest age-validated freshwater fish^11^.

We investigated the relationship between age and multiple physiological systems that are expected to be important mechanisms of senescence including: an aspect of the stress response (neutrophil to lymphocyte ratio, henceforth NLR), immune function, and telomere length. In many organisms, the stress response becomes disregulated with age. NLRs are often reflective of long-term chronic stress exposure, increase with age in vertebrates^13–16^, and are negatively related to several fitness parameters such as increased susceptibility to disease^17^, slower growth rates^18^, and lower survival to the next breeding season^19^ in birds. The immune system also often declines with age in humans and other animals^20,21^. In free-living Soay sheep, resistance to infection declines with age and results in decreased probability of surviving the winter^10^. Telomeres are conserved, repetitive sections of non-coding DNA at chromosome ends that enhance genome stability, but shorten during cell replication^22^ and in response to stress^23–25^. Telomeres limit cellular lifespan because once they become critically short, cell replication and function terminates, which is expected to increase organismal aging^26^. Telomere length is negatively correlated with age in diverse species^27–32^ and individuals with longer telomeres often have greater longevity^33,34^.

In this study, we examined the relationships between age, NLR, immune function, and telomere length across the lifespan in bigmouth buffalo to gain novel insight into the progression of senescence in several physiological systems in this extremely long-lived vertebrate.

## Results

In total, we sampled 240 fish ranging in age from 2 to 102 years old, with a median age of 15 years. Age distributions differed across sites (see Lackmann et al*.*^11^), which we briefly summarize. Fish collected from Artichoke Lake (range 2–43 years old, median 5 years old) and Otter Tail River (3–80 years old, median 13 years old) had similar age distributions (and similar to the overall distribution). Whereas fish from the Lake Minnetaga (2–15 years old, median 4 years old) site were younger, perhaps due to collection bias by the commercial harvesters, and fish from the Pelican Lakes (32–102 years old, median 85 years old) sites were older, possibly due to a lack of recruitment over the last several decades.

**Figure 1:**
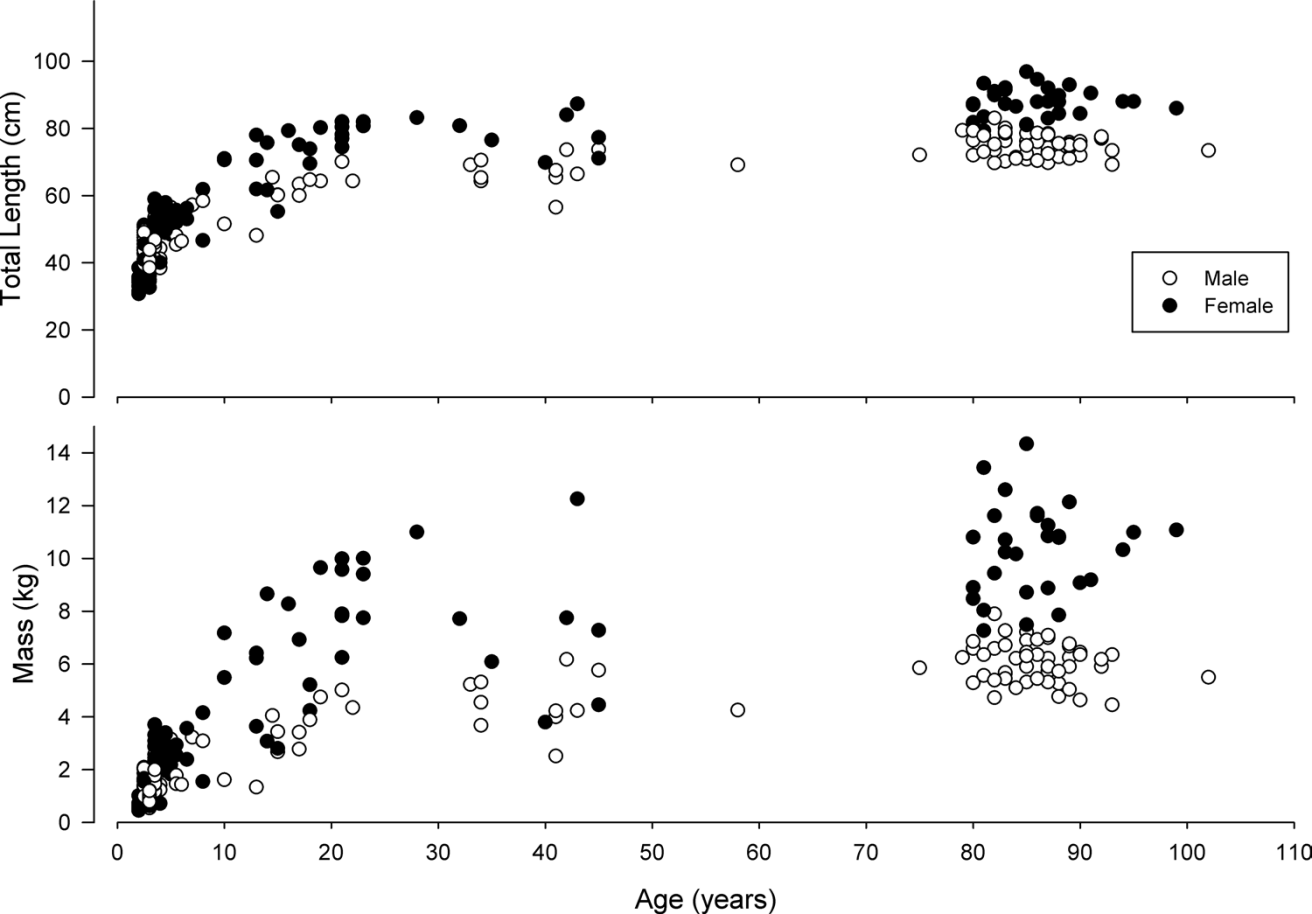
Age and size (total length above, mass below) of bigmouth buffalo (*Ictiobus cyprinellus*). Open circles represent males and filled circles represent females.

The length of the fish ranged from 30.7 to 96.9 cm (median 60.1 cm) and masses ranged from 0.45–14.33 kg (median 3.42 kg), with females reaching larger sizes for a given age than males (Lackmann et al*.*^11^) (Fig. 1). Fish collected from the Artichoke Lake (30.7–87.3 cm, median 47.9 cm; 0.45–12.25 kg, median 1.77 kg), Lake Minnetaga (39.5–65.4 cm, median 49.6 cm; 0.98–4.04 kg, median 1.98 kg) and Otter Tail River (36.1–87.3 cm, median 56.5 cm; 0.54–8.47 kg, median 2.67 kg) sites had similar size distributions (and similar to the overall distribution). However fish from the Pelican Lakes (64.3–96.7 cm, median 76.5 cm; 3.67–14.33 kg, median 6.44 kg) site were larger. An orthogonal regression indicated log-transformed total length was significantly correlated to log-transformed mass (for females: ln total length = 1.35 + 0.34 * ln mass, R^2^ = 0.99, n = 128; for males: ln total length = 1.37 + 0.34 * ln mass, R^2^ = 0.98, n = 127), and residuals from these regressions quantified condition. We included all data that was collected for each dependent variable.

### Age and a measure of stress

**Figure 2:**
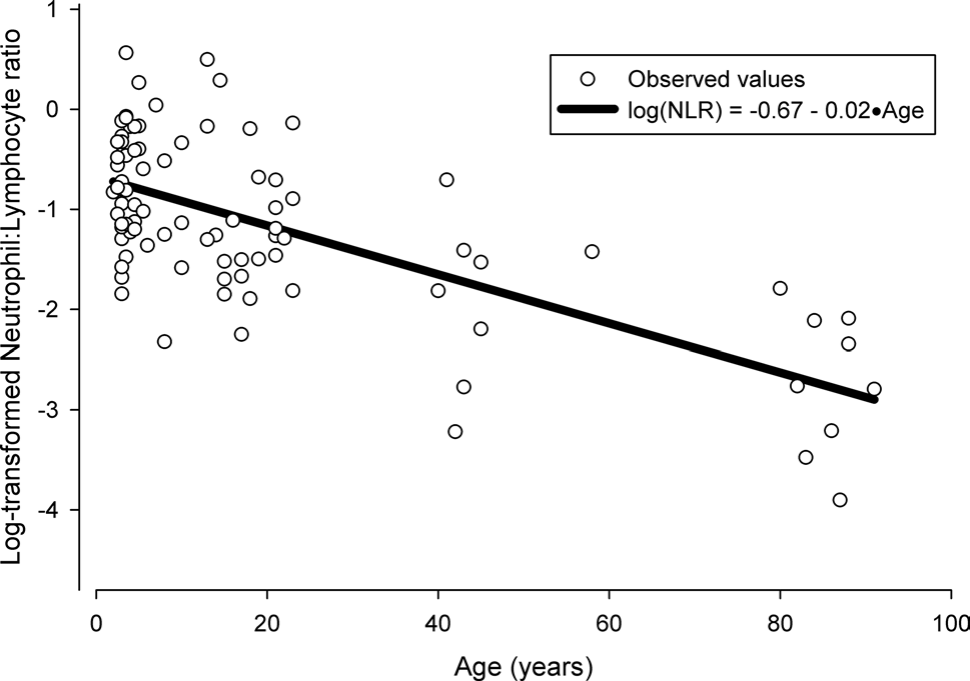
The relationship between age and log-transformed NLR in bigmouth buffalo (*Ictiobus cyprinellus*). NLR significantly decreased with age (F_1,86_ = 78.26, p < 0.01, r^2^ = 0.48).

We measured NLR in 92 individuals ranging in age from 2 to 91 years old, and linear models with age and site explained significant amounts of variation in NLR. In a linear regression with data from all sites combined (Methods contains details on analysis), there was a significant negative relationship between age and NLR such that age explained approximately 48% of the variation in log-transformed NLR (F_1,86_ = 78.26, p < 0.01, r^2^ = 0.48) (Fig. 2). A regression model including data from only the Artichoke Lake and Otter Tail River sites (which have populations with similar age distributions that span the range of the ages from all sites) also indicated a significant negative relationship between NLR and age (F_1,57_ = 11.07, p < 0.01, r^2^ = 0.16). The ANOVA of NLR by site indicated that the Pelican Lakes site (i.e., population) had significantly lower NLR (F_3,88_ = 24.71, p < 0.01, r^2^ = 0.46) than the other sites (i.e., populations). The Pelican Lakes site lacks individuals less than 32 years old (potentially skewing age effects seen in the overall model), but a post hoc model including data from the three other sites (i.e., Artichoke Lake, Lake Minnetaga and Otter Tail River) also indicated a significant negative relationship between age and NLR (F_1,77_ = 17.79, p < 0.01, r^2^ = 0.19). Total length (F_1,90_ = 27.3, p < 0.01 r^2^ = 0.23) had a significant negative relationship with NLR, condition (F_1,90_ = 8.54, p < 0.01, r^2^ = 0.09) had a significant positive relationship with NLR, and sex did not explain any of the variation (F_1,90_ = 0.00 p = 0.95, r^2^ < 0.01) in NLR.

### Age and an aspect of immunity

**Figure 3:**
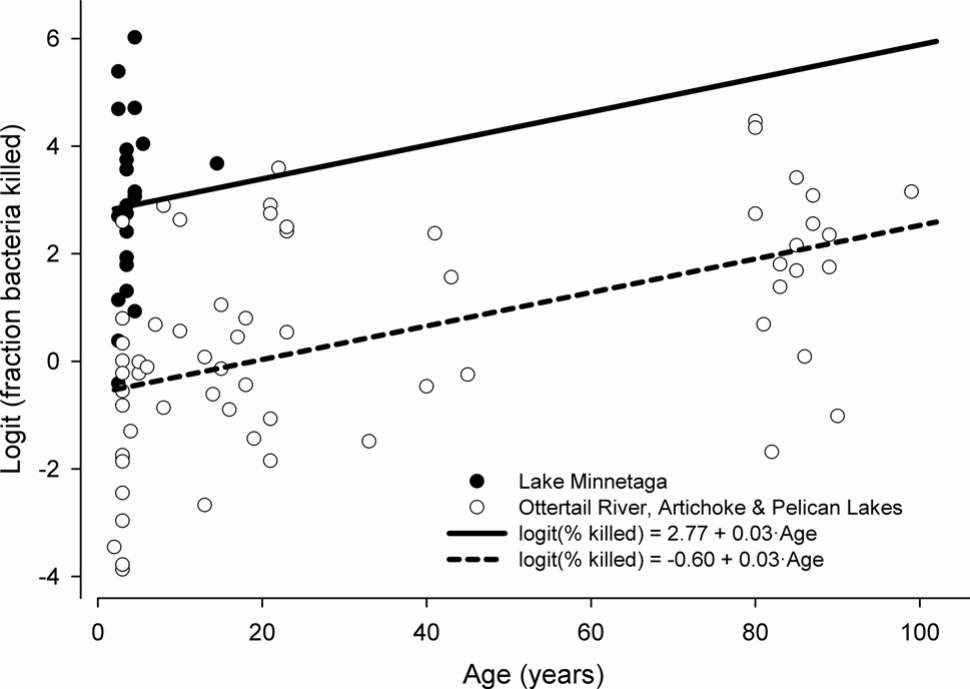
The relationship between age and bacterial killing capacity (logit-transformed) in bigmouth buffalo (*Ictiobus cyprinellus*) from Lake Minnetaga (filled circles, solid line) and Artichoke Lake, Ottertail River and Pelican Lakes (open circles, dashed line) with regression lines from the post hoc model with additive effect of site and age. Bacterial killing capacity significantly increased with age (F_1,85_ = 23.29, p < 0.01, r^2^ = 0.17).

Bacterial killing capacity was measured in 86 individuals ranging in age from 2 to 99 years old, and killing capacity increased with age, was greater in individuals from Lake Minnetaga, and was greater in males. In the regression model with data from all sites combined, age did not explain variation in bacterial killing capacity (F_1,84_ = 13.18, p = 0.10, r^2^ = 0.03). A regression model with data from all sites combined including terms for both age and sample date did explain 8% of the variation in bacteria killing capacity (F_2,83_ = 3.41, p = 0.04, r^2^ = 0.08) but only the date term was significant (F_1,83_ = 3.96, p = 0.05, r^2^ = 0.04). However in the linear regression including data from the Artichoke Lake and Otter Tail River sites (which have populations with similar age distributions that span the range of the ages from all sites), bacterial killing capacity was significantly positively related to age (F_1,46_ = 14.75, p < 0.01, r^2^ = 0.25). Furthermore, the linear regression including data from the Artichoke Lake and Otter Tail River sites including both age and sample date terms, approximately 26% of the variation in bacterial killing capacity was explained (F_2,44_ = 7.83, p < 0.01, r^2^ = 0.26) but only the age term (F_1,44_ = 11.29, p < 0.01, r^2^ = 0.19) was significant (Date term: F_1,44_ = 0.93, p = 0.34, r^2^ = 0.02). Site explained a significant amount of the variation in killing capacity (F_3,85_ = 15.83, p < 0.01, r^2^ = 0.36) (age estimates could not be obtained for two individuals from Lake Minnetaga) in the ANOVA, because individuals from Lake Minnetaga had significantly higher bacterial killing capacity than fish from the other sites combined (F_1,87_ = 30.11, p < 0.01, r^2^ = 0.26). Because known-age individuals from Lake Minnetaga were all younger than 15 years, we used a post hoc ANOVA to compare bacterial killing capacity in Lake Minnetaga individuals to individuals less than 15 years old from the other sites combined (thereby removing any effects resulting from different age distributions), and again found that individuals from Lake Minnetaga had significantly higher killing capacity (F_1,49_ = 51.72, p < 0.01, r^2^ = 0.51). In a model restricted to individuals from the Artichoke Lake, Otter Tail River and Pelican Lakes sites (thereby removing the Lake Minnetaga site effect), the significant positive relationship between age and bacterial killing capacity was again observed (F_1,62_ = 22.07, p < 0.01, r^2^ = 0.26). Similarly, for the model restricted to individuals from the Artichoke Lake, Otter Tail River and Pelican Lakes sites including a sample date term, the significant positive relationship between age and bacterial killing capacity was again observed (Full model: F_2,61_ = 12.48, p < 0.01, r^2^ = 0.29; Age term: F_1,61_ = 21.42, p < 0.01, r^2^ = 0.25) but the date effect was not significant (F_1,61_ = 2.40, p = 0.13, r^2^ = 0.03). Finally a post hoc model in which sites were grouped by Lake Minnetaga versus other sites combined and including an age effect explained more than 40% of the variation in bacterial killing capacity (F_2,83_ = 28.54, p < 0.01, r^2^ = 0.41) (Fig. 3), with both a significant site effect (F_1,85_ = 52.65, p < 0.01, r^2^ = 0.38) and a significant increase with age (F_1,85_ = 23.29, p < 0.01, r^2^ = 0.17). A similar post hoc model with effects of site (Lake Minnetaga versus other sites combined), age and sample date (F_3,82_ = 20.19, p < 0.01, r^2^ = 0.42) also showed only significant effects of site (F_1,82_ = 49.76, p < 0.01, r^2^ = 0.35) and age (F_1,82_ = 22.50, p < 0.01, r^2^ = 0.16) but not date (F_1,82_ = 2.48, p = 0.12, r^2^ = 0.02).

Total length (F_1,87_ = 1.21, p = 0.27, r^2^ = 0.01), mass (F_1,87_ = 0.23, p = 0.63, r^2^ < 0.01), and condition (F_1,87_ = 0.01, p = 0.91, r^2^ < 0.01) did not explain significant amounts of the variation in bacterial killing capacity, but males had slightly greater killing capacity (F_1,87_ = 3.99, p = 0.04, r^2^ = 0.04). There was a significant negative correlation between telomere length and bacterial killing capacity (R = −0.27, n = 70, p = 0.03). NLR was not significantly correlated with bacterial killing capacity (R = 0.15, n = 59, p = 0.25).

### Age and telomere length

**Figure 4:**
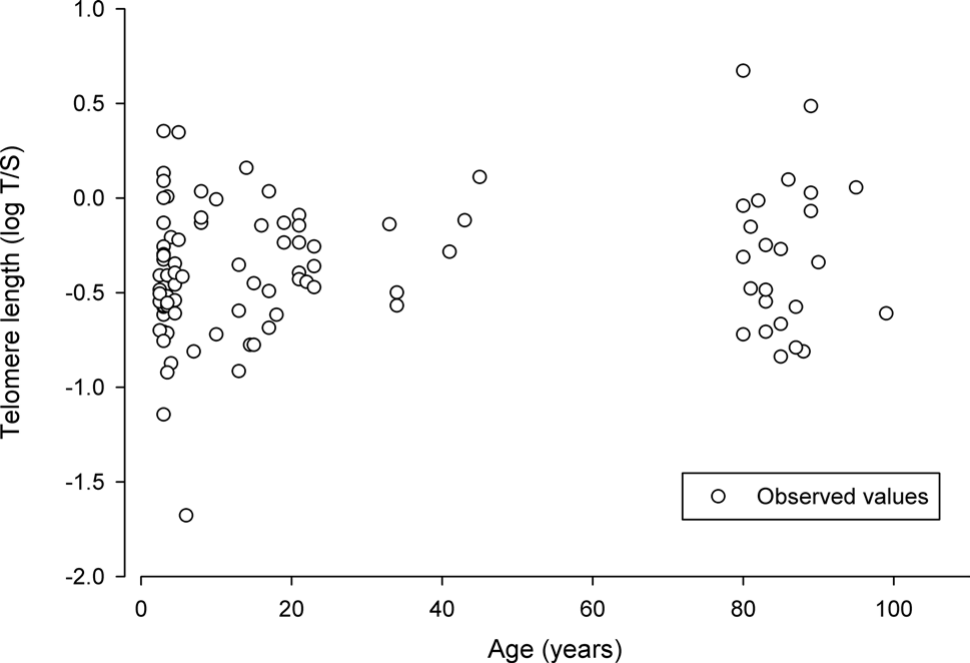
The relationship between age and relative telomere length measured from red blood cells (expressed as the natural log of the T/S ratio) of bigmouth buffalo (*Ictiobus cyprinellus*). There was no significant relationship between age and telomere length (F_1,95_ = 0.65, p = 0.42, r^2^ = 0.01).

Relative telomere length was measured in 97 individuals ranging in age from 2 to 99 years old, and variation in telomere length was not related to age, site, sex, size or condition. In a linear regression with data from all sites combined, age did not explain any of the variation in telomere length (F_1,95_ = 0.65, p = 0.42, r^2^ = 0.01) (Fig. 4). Furthermore, the linear regression with data from the Artichoke Lake and Otter Tail River sites (which have populations with similar age distributions that span the range of the ages from all sites) indicated that age did not explain any of the variation in telomere length (F_1,50_ = 0.23, p = 0.63, r^2^ < 0.01). Finally, site did not explain a significant amount of the variation in telomere length (F_3,93_ = 2.31, p = 0.08, r^2^ = 0.08). Telomere length was significantly negatively correlated with NLR (R = −0.27, n = 63, p = 0.03). Total length and mass, respectively, did not explain a significant amount of the variation in telomere length (F_1,95_ = 0.72, p = 0.40, r^2^ = 0.01) (F_1,95_ = 1.58, p = 0.21, r^2^ = 0.02). Sex and condition also failed to explain a significant amount of variation in telomere length (F_1,95_ = 0.05, p = 0.82, r^2^ = 0.00) (F_1,95_ = 1.09, p = 0.30, r^2^ = 0.01).

### Breusch-Pagan tests

To determine whether the variation in these traits changed with age, which could be a signature of past selection, we conducted Breusch-Pagan tests. Breusch-Pagan did not indicate that there was heteroscedasticity in NLR (F_1,86_ = 0.51, p = 0.47, r^2^ = 0.01), bacterial killing (F_1,84_ = 1.07, p = 0.77, r^2^ < 0.01; based on regression model shown in Fig. 3), or relative telomere length (F_1,95_ = 0.41, p = 0.52, r^2^ = 0.00) across the age gradient, indicating that the variance in these traits did not decrease with age.

## Discussion

The physiological systems that decline with age and contribute to variation in senescence are not well understood. Interestingly, our results are consistent with the idea that several physiological measures improve, rather than decline with age in this extremely long-lived fish. We found that older individuals have lower NLRs, better immune function, and do not show any evidence of telomere shortening. These results contradict many of the age-related patterns of senescence that have been observed in other vertebrates and may suggest a negligible rate of senescence in bigmouth buffalo. An important caveat is that our findings are based on cross-sectional data rather than longitudinal sampling within individuals across years. Consequently, cohort effects and differential survival of high quality individuals may have contributed to these results. However, if these patterns were due to past selection events, variation in these physiological traits would be expected to decline with age. Yet, we did not find any evidence for this as the Breusch-Pagan tests indicated that variance in NLR, immune function, and telomere length did not differ with age. Thus, we think it is unlikely that our results are simply due to cross-sectional sampling.

Many of the observations we made in bigmouth buffalo contradict the age-related patterns observed in other vertebrates. For example, NLR often increases with age, possibly because the ability to cope with stressors becomes disregulated^13–16^. This has been demonstrated in birds where low lymphocyte counts are associated with increased susceptibility to disease, slow growth, and decreased survival^17–19^. However, we found that older individuals had lower rather than higher NLRs than younger individuals (Fig. 2), suggesting that older individuals experience lower levels of chronic stress and may have a more efficient stress response and even greater fitness than younger individuals.

Older individuals also failed to display the age-related decline in immune function that is well known in mammals ^35^. Instead, our findings in bigmouth buffalo indicate that senescence of the immune system is not observed in individuals of 90 + years old, as killing capacity actually *increased* with age (Fig. 3). The impact that age has on the immune system of ectotherms is not fully understood and, unlike mammals, temperature influences immune function in ectotherms^36^. Consistent with our results, long-lived turtles do not show evidence of immunosenescence^35,37^ and water pythons (*Liasis fuscus*) appear to maintain immunocompetence with age by using natural antibodies^38^. In contrast, relatively short-lived lizards do show a strong decrease in immune function with age^39^. More research is needed to better understand how variation in life-history strategies influence age-related patterns in the immune systems of long-lived ectotherms. Although we only measured one aspect of innate immunity, our results clearly indicate that it does not decline in bigmouth buffalo even into old age.

Not only did we observe evidence of lower chronic stress and more efficient immune function, we also found no evidence that telomere length declines with age in this species (Fig. 4). Telomere length shortens with age in humans and many other mammals and birds^27–32^, and critically short telomeres are thought to lead to increased organismal aging and eventual mortality^26^. Although less well understood, recent studies suggest that the telomere dynamics of endotherms and ectotherms may differ, especially since ectotherms may experience temperature dependent changes in telomere processes^40^. For example, there is evidence that telomerase, a reverse transcriptase enzyme that can maintain and restore telomeres^41^, continues to be expressed in somatic tissues into adulthood in some ectotherms^40^. In fact, unlike mammals, high telomerase activity has been observed in all life stages of fishes^42–45^ and in zebrafish (*Danio rerio*) telomerase levels and telomere length are closely related throughout the entire life cycle^42^.

Previous research on telomere dynamics in fishes has yielded mixed results on the relationship between age and telomeres^42–49^(see review in Simide et al*.*^49^). However prior to our study, almost no information was available on telomere dynamics in fish over 20 years old. Two studies have examined telomere length in fish with a lifespan greater than 20 years and the results are inconclusive^49^_._ In one of these studies, common carp (*Cyprinus carpio*) that were larger (and presumably older) did not have shorter telomeres^46^. Common carp and bigmouth buffalo are in the same order (Cypriniformes) and have come to live in similar habitats in North America, and share some similarities in life history^50^. Thus, our findings in bigmouth buffalo are consistent with those in a similar long-lived teleost^46^. Currently it is unknown whether bigmouth buffalo continue to express telomerase throughout life and measuring telomerase levels within individuals is needed to determine if telomerase plays a significant role in determining telomere length in bigmouth buffalo and other long-lived fishes.

Although telomere length was not correlated with age, individuals that had higher NLRs, and had likely experienced more stress exposure or were more sensitive to it, had shorter telomeres. Chronic stress is associated with shorter telomere length in vertebrates^23–25,51–55^, as demonstrated by a similar pattern observed in two species of long-lived seabirds, where telomere loss was not related to age but appeared to be impacted by early life events^55^. In the long-lived Siberian sturgeon (*Acipenser baerii*), heat stress significantly reduced telomere length after only one month of exposure^49^. Extrinsic factors such as environmental stress may play a larger role in telomere dynamics in bigmouth buffalo and other long-lived fishes, especially if these species have mechanisms for preventing telomere shortening (e.g., telomerase production).

According to the disposable soma theory of aging, organisms must allocate a finite amount of resources among competing life-history traits to maximize fitness, and investment in growth and reproduction will necessarily come at a cost to investment in self-maintenance^56^. Bigmouth buffalo appear to continue to invest in immunity and somatic maintenance long after sexual maturation. But, this life-history strategy is expected to come at a cost to investment in reproduction^56^. However, in indeterminate growers, there may be advantages to investing into longevity. In these species, adults that have protective morphology (i.e., large size) and indeterminate fecundity should increase their lifetime fitness if they continue to successfully and increasingly reproduce throughout adulthood^5^. In this case, selection for mechanisms that can extend longevity can be strong^5^. This life-history strategy is particularly likely in species that exhibit low extrinsic mortality, indeterminate growth, and increasing fecundity with size^5^, making bigmouth buffalo a prime candidate as old individuals lack any natural predators and produce increasingly large numbers of viable gametes. The bigmouth buffalo reproductive strategy may rely on repeated attempts at reproducing throughout life, especially when an individual is larger and more fecund, to take advantage of fortuitous seasons for offspring recruitment that may occur infrequently^57^. Accordingly, continual investment into self-maintenance (i.e., stress response, immunity, and somatic tissue) may be a life history strategy by which bigmouth buffalo increase their overall fitness.

In contrast to terrestrial vertebrates, we found no evidence of age-related physiological deterioration in several physiological systems in bigmouth buffalo, even in individuals approaching 100 years old. NLR decreased with age, immune function improved with age, and telomere length was not correlated with age, all observations that contradict the typical path of age-related declines observed in humans and other vertebrates. Finch^12^ categorized senescence into three categories based on rapid, gradual, or negligible rates of progression. While rapid and gradual senescence were readily accepted concepts, Finch’s hypothetical notion of negligible senescence remains controversial^58^. Finch and Austad^59^ described the criteria for negligible senescence to include a lack of age-related increase in mortality or decrease in reproduction, as well as a lack of age-related decline in physiological capacity or disease resistance. Thus, our data are consistent with these criteria for negligible senescence as we observed many old, reproductive individuals with long telomeres, high condition values, and relatively efficient functioning stress responses and immune systems. An important caveat is that this a cross-sectional study, which can obscure age-related changes within individuals. Longitudinal studies are necessary to further clarify the process of physiological senescence in this species, but collecting samples over the required time frame is likely to be logistically challenging. Future research on other extremely long-lived organisms will be critical for understanding the mechanisms that underlie variation in pace of senescence.

## Materials and methods

### Study species and sample collection

Bigmouth buffalo are native to the Mississippi and Hudson Bay drainage basins from Canada to the Gulf of Mexico^60^, inhabiting shallow lakes and slow-moving rivers^50^. Individuals may migrate up to hundreds of kilometers in unfragmented waters^61^, however the systems in this study are highly fragmented by dams^11^. They breed once annually in the spring while age at first reproduction occurs around 8–9 years for females and 5–6 years for males^11^. The main causes of mortality in bigmouth buffalo are unknown, however it is likely that predation drives mortality in young individuals and that the commercial and recreational harvests play a significant role in adult mortality^11,62^.

We collected bigmouth buffalo (*Ictiobus cyprinellus*) in Minnesota from four sites within the Red River and Mississippi River basins. Between 2017–2018, fish were obtained from the following four sites: 1) Artichoke Lake (n = 52, date of collection May 4th 2017) and 2) Lake Minnetaga (n = 66, September 22nd, 2017) in the Mississippi River basin and from 3) Otter Tail River (just downstream of Orwell Dam) (n = 33, April 7th 2018) and 4) Lake Lizzie (August 5th–September 2nd 2017), Pelican Lake ( July 28th 2017), Rush Lake (May 12th 2018), and North Lida Lake (August 3rd 2017) in the Red River basin. Lakes Lizzie, Pelican, Rush and North Lida are geographically close, interconnected lakes along the Pelican River, and because fish movement among these lakes is possible we considered them a single site (Pelican River Lakes (n = 89)) in analyses. The Lake Minnetaga and Artichoke Lake populations receive significant commercial harvest, and the Pelican River Lakes population has had sporadic successful recruitment, if any, over the past 80 years^11^.

Fish were collected via hook and line anglers, the recreational bowfishing harvest (bow and arrow), and the commercial harvest (seine nets). All animals were treated in accordance with North Dakota State University’s guidelines for animal care, and all procedures were conducted in accordance with animal protocol A17007 approved by the North Dakota State University Institutional Animal Care and Use Committee. This study complies with the ARRIVE guidelines. Immediately following collection, total length (± 0.1 cm) and wet mass (± 0.1 kg) were recorded from each fish. Following measurement, fish were euthanized by overanesthetizing with tricaine methanesulfonate and a blood sample (approximately 3 mL) was obtained from a gill arch and placed in a heparinized container (BD Vacutainer). Following blood collection, whole fish carcasses were placed on ice and returned to the lab at North Dakota State University within three hours and frozen at − 20 °C. Blood samples were also placed on ice and returned to the lab, where a small drop of whole blood was smeared on a glass microscope slide for each sample. Samples were then centrifuged (1700 G for 10 min) for plasma separation. Individual plasma samples were removed with a pipette and placed in 1.5 mL Eppendorf Tubes, and the packed red blood cells were left in the original BD Vacutainer, then red blood cell and plasma samples were frozen (− 20 °C) until later analysis. Blood smears were allowed to air dry, then stained with a Hemacolor staining kit.

### Dissection

Carcasses were dissected to determine sex and collect otoliths for age determination. Otoliths are calcium carbonate structures within the inner ear of fish that display annual rings of growth associated with seasonality in water temperature. As many otoliths as possible were extracted from each fish by cutting into the cranium and first vertebral column from the ventral side of the fish and removing the labyrinth organ. Otoliths were extracted from the labyrinth organ and stored at room temperature in 1.5 mL Eppendorf Tubes filled with water prior to preparation for age analysis.

We obtained age estimates from counts of annuli in thin-sectioned otoliths, and ages were validated using bomb radiocarbon dating^11^. In brief, otoliths were dissected from the cranium of each fish, embedded in epoxy, and thin-sectioned using a Buehler IsoMet 1000 saw with twin blades^63^. Thin sections of the otoliths were photographed at 75X under a compound microscope and images were examined for annuli. Otolith sections were scored by multiple readers, with consensus readings used to determine the final age assigned to each specimen (see Lackmann et al*.*^11^ for more details regarding the age-reading protocol).

Assigned ages were validated by bomb radiocarbon dating, which is preferred for validating the ages of long-lived fish^64^. Otolith age scores were validated both cross-sectionally and longitudinally, and multiple otolith types were used to determine the age of specimens (see Lackmann et al*.*^11^).

### NLR analysis

The effects of glucocorticoid stress hormones on white blood cell production are conserved across taxa and white blood cell enumeration is often used as a quantitative measure of stress exposure in vertebrates (reviewed by Davis et al*.*^14^). An increase in glucocorticoids in response to stress leads to an increase in the ratio of neutrophils to lymphocytes (NLR) within individuals^14^. However, NLR can be used as an indicator of long-term stress as it remains elevated (following a stressor) much longer compared to glucocorticoids^14^. The time lag associated with the white blood cell response to stress is also longer, especially in ectothermic animals, eliminating concerns of the stress caused by handling and capture^14^. Neutrophils and lymphocytes can be easily identified in fish blood smears, and the patterns observed relating to stress are identical to those seen in other vertebrates^14^. We obtained counts of leukocytes from the stained blood smears. Smears were examined under a compound microscope (400× magnification), and neutrophils and lymphocytes were counted until the combined total count exceeded 100^65^. NLR was calculated by dividing the number of neutrophils by the number of lymphocytes. We also recorded the number of microscope viewing fields required to achieve the 100 cell count for the NLR. Quantification of NLR (and all dependent variables) was blinded to the size, age, or other characteristics of individuals.

### Immune function analysis

We quantified immune system strength using a bactericidal assay. This assay assesses the ability of the innate immune system to eliminate an actual pathogen and involves the use of phagocytes, opsonizing proteins, and natural antibodies^66^. We used a bactericidal assay described by Zysling et al.^67^ with the following modifications: we used plasma instead of whole serum, measured each sample in triplicate, and adjusted concentrations to yield control plates with approximately 250 bacterial colonies. Briefly, a working solution of *E. coli* (Epower Microorganisms #0483E7, ATCC 8739, Microbiologics, St. Cloud, MN) was mixed with serum that was diluted with CO2-independent media. This solution was activated by incubation at 37 °C for 30 min. Fifty microliters (50 µl) of solution was then plated on tryptic soy agar plates and allowed to incubate overnight. Control plates were created by diluting the working solution with media alone. Colonies were counted on each plate and the mean number of colonies on sample plates was divided by the mean number of colonies on control plates. This fraction was subtracted from 1 and multiplied by 100 to express killing capacity as the percentage of bacteria killed relative to control plates.

### Telomere length analysis

We determined relative telomere length from DNA extracted from red blood cells. Genomic DNA was extracted from red blood cells using a Nucleospin Blood kit (Macherey–Nagel, Inc.). The concentration of extracted DNA was measured using a NanoDrop spectrophotometer and only DNA extractions with full integrity (determined by gel electrophoresis) were utilized in telomere length analysis. Relative telomere length was measured using quantitative PCR (qPCR) on an Mx3000P qPCR system (Stratagene, Cheshire, UK). We followed the methods of Heidinger et al*.*^68^ with slight modifications for this species. Beta-actin primers (from the available genome of *Myxocyprinus asiaticus*, a species in the same family as bigmouth buffalo) were used as the control, single copy gene. The suitability of our control, single copy gene was tested by a melt curve analysis, which established that the dissociation curve had a single peak. Telomere and beta-actin reactions were run on separate plates. Reactions were run in duplicate. Telomere length was calculated as the ratio (T/S) of telomere repeat copy number (T) to control, single gene copy number (S) of the focal sample relative to a reference sample^68^. Telomere lengths were calculated across three microplates. Intra-plate variation in cycle threshold (Ct) values among duplicates was calculated for all plates. An arbitrary, single sample was run on all plates to allow for the calculation of inter-plate variation of T/S ratios that were run on three separate plates. A water sample was included on every plate as a negative control. Every plate also included a sample from the same single individual that was serially diluted to produce a standard curve, and used to measure reaction efficiencies. Average reaction efficiencies were 95.9 ± 1.67% (mean ± 1 SEM) and 101.0 ± 0.87% for beta-actin and telomere microplates, respectively. For telomere measurements, the inter-plate coefficient of variation for the repeated sample T/S ratios was 11.0%. Reactions were highly replicable, with the average coefficient of variation between replicate Ct values equal to 0.71% for telomere measurement plates and 0.26% for beta-actin plates. The components of qPCR reactions were: 12.5 µL of SYBR Green Master Mix, 0.25 µL of forward and reverse primers, 6 µL of water, and 24 ng of DNA diluted in 6 µL of water, totaling to 25 µL. The conditions for the qPCR reactions were: telomeres 10 min at 95 °C, followed by 27 cycles of 15 s at 95 °C, 30 s at 58 °C, and 30 s at 72 °C; b-actin 10 min at 95 °C, followed by 40 cycles of 15 s at 95 °C, 30 s at 57 °C, and then 30 s at 72 °C. For both reactions, the number of PCR cycles (Ct) required to create sufficient fluorescent signal to cross a threshold was measured. Individuals with relatively long telomeres were characterized by shorter reaction times^68^.

### Statistical analysis

To examine the potential effects of age on NLRs, immune function, and telomere length, we used general linear models that included age, site, size and condition as fixed effects and NLR, bacterial killing capacity, and telomere length as covariates. Telomere lengths and NLRs followed a log-normal distribution, so we log-transformed both for statistical analysis. Killing capacity was logit-transformed. We obtained residuals (on the mass axis) from an orthogonal regression of log-transformed mass and log-transformed total length of males and females separately, and refer to the residual as condition^69^. We analyzed relationships among NLR, killing capacity and telomere length (i.e., the dependent variables) using Pearson’s correlation coefficient. Age distributions differed among sites (see Lackmann et al*.*^11^, but also summarized in the Results), and limited our ability to detect synergistic effects between age (our primary interest) and site. Therefore we conducted a series of analyses using data across all sites, then restricted to sites with overlapping age distributions. For each dependent variable (i.e., NLR, bacterial killing capacity, telomere length), we first used a linear regression to look for effects of age for samples from all sites. Second, we repeated the linear regression for samples restricted to the Otter Tail River and Artichoke Lake sites (which have overlapping age distributions). Third, we used an ANOVA to test for site effects using samples from all sites. If a site effect was detected, we repeated the ANOVA but restricted the analysis to age ranges that were present at each site. Finally, if a site effect was detected we reanalysed the linear regression with age excluding samples from the differing site. We assumed statistical significance at α = 0.05. For each linear regression using age, we also examined a regression with a term for age^2^ in case there were nonlinear age effects (e.g., high levels of NLR at young and old ages but low levels at intermediate ages), but the effects of the age^2^ term were not significant (i.e., α > 0.05) in any cases and we do not present those results for conciseness. Similarly we included a term for date in each linear regression with age, but only found effects in a single instance (for bacteria killing capacity) and have only reported those results for conciseness. We used the Breusch-Pagan test to check for equal variance (i.e., homoscedasticity) in regressions of NLR, killing capacity, and telomere length with age. All statistical analyses were conducted using JMP 13 for Windows (SAS Institute Inc., Cary, NC, USA).

## Data Availability

The data that support the findings of this study will be made available on Dryad upon acceptance.
